# Environmental DNA (eDNA) Technology in Biodiversity and Ecosystem Health Research: Advances and Prospects

**DOI:** 10.1002/ece3.72891

**Published:** 2026-01-09

**Authors:** Shuwen Wu, Yun Wang, Haiyan Qin, Zeyu Zhang, Shijun Liu, Yunjie Ruan, Guangsuo Chen, Xia Yuan, Hangjun Zhang

**Affiliations:** ^1^ Hangzhou Normal University Hangzhou China; ^2^ Zhejiang Provincial Key Laboratory of Wetland Intelligent Monitoring and Ecological Restoration Hangzhou China; ^3^ The Rural Development Academy, College of Bio‐Systems Engineering and Food Science Zhejiang University Hangzhou China; ^4^ Hangzhou Zhijun High Technology Company Limited Hangzhou China

**Keywords:** biodiversity, ecosystem health, environmental DNA, monitoring and assessment

## Abstract

Environmental DNA (eDNA) technology, as a minimally invasive or noninvasive monitoring approach, has been increasingly applied in biodiversity surveys and ecosystem health assessment by detecting genetic material in environmental samples. This approach exhibits high sensitivity for identifying rare, endangered, and invasive species, with broad applicability across aquatic, terrestrial, and atmospheric ecosystems. Moreover, eDNA metabarcoding enables large‐scale detection of microbial community structure and function. By systematically synthesizing multi‐environment case studies, this review evaluates optimized eDNA workflows, including sampling (0.22–0.45 μm filtration for aquatic systems, PCI/DNeasy methods for soils, and MD8 samplers for air), DNA extraction, and bioinformatic analysis, integrating standardized guidelines to enhance research reproducibility and comparability. Despite advantages such as reduced field labor and cost efficiency, eDNA applications still face critical challenges, such as reference database gaps, full‐process quality control risks, methodological inconsistencies, and limitations in abundance quantification. Future advancements in sequencing technologies, bioinformatics, and interdisciplinary integration (machine learning, remote sensing) are expected to expand eDNA's role in tackling global change issues such as climate adaptation, pollution tracking, and ecological restoration.

## Graphical abstract

This review synthesizes over 15 years of advancements in eDNA technology by systematizing standardized guidelines for sampling, extraction, and bioinformatics, thereby enhancing the reproducibility of eDNA‐based research. It highlights eDNA's transformative role in noninvasively detecting rare, endangered, and invasive species, as well as its value in ecosystem health assessment, while also addressing critical challenges facing eDNA applications, including methodological inconsistencies, limitations in species abundance quantification, and contamination risks to broaden the technology's field utility. Additionally, the review explores future advancements in eDNA research, focusing on the integration of sequencing technologies, bioinformatics, and machine learning to further expand eDNA's application in climate adaptation and ecological monitoring.

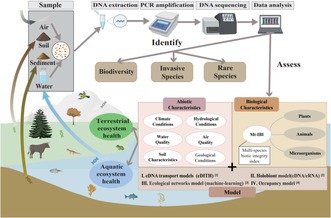

## 1 Introduction

Biodiversity encompasses the variety of life, across genetic, species to ecosystems levels, reflecting the structure, function, distribution, and composition of all living organisms (Skidmore et al. 2021). The biodiversity crisis has escalated from a local concern to a global priority, as evidenced by international initiatives such as the United Nations Sustainable Development Goals (SDGs), the Aichi Targets, ongoing post‐2020 negotiations of the Convention on Biological Diversity (CBD), and the risk assessments conducted by the Intergovernmental Science‐Policy Platform on Biodiversity and Ecosystem Services (IPBES) (Bongaarts 2019). Such global attention is warranted because accelerating environmental changes and persistent biodiversity loss are increasingly compromising critical ecosystem functions and services (Altermatt et al. 2025; Oliver et al. 2015). Therefore, robust assessment indicators and methods are needed to detect ecological issues, guide conservation strategies, and promote sustainable management (Zong et al. 2024). However, traditional approaches to biodiversity and ecosystem health assessment have apparent inadequacies, such as being time‐consuming and labor‐intensive, limiting the spatial extent and temporal resolution of monitoring (Johnston et al. 2023). In response, environmental DNA (eDNA) technology has emerged as a minimally invasive and effective approach for biodiversity evaluation and ecosystem health assessment, offering enhanced efficiency and accuracy (Takahashi et al. 2023; Kim, Cho, Kim, et al. 2024).

Environmental DNA is genetic material originating from the hair, skin, urine, feces, gametes, or carcasses of organisms that is present, in a more or less degraded form, in water, soil, or air (Beng and Corlett 2020). This material is analyzed using DNA sequencing techniques, including Sanger sequencing, chemical cleavage, and next‐generation sequencing (NGS), to characterize or quantify target organisms, thereby determining their distribution and functional roles within ecosystems (Miya 2022). Typically, DNA barcoding is a method of specimen identification using short, standardized segments of DNA, which is a foundational technique that creates a “species genetic ID card” by sequencing specific gene fragments of an organism. In contrast, eDNA technology represents a comprehensive research strategy that uses DNA from environmental samples and relies on molecular biology techniques, such as DNA barcoding as its core analytical tool, enabling biodiversity assessment and ecosystem monitoring at a higher dimension (e.g., community level) (Cristescu 2014). The concept of eDNA was first introduced in 1987 by Ogram, who studied microbial DNA extraction and purification in sediments (Ogram et al. 1987). However, it was not widely recognized and applied by ecologists until after 2000, driven by the advancement of molecular technologies. Compared to traditional survey methods, eDNA technique offers several key advantages, foremost among which is its minimally invasive or noninvasive characteristic. Additionally, eDNA analysis does not depend on specialized special equipment or specific observation times, making it particularly useful for detecting species that are difficult to observe due to their small size or low numbers (Sun et al. 2024). Moreover, it exhibits high sensitivity, time efficiency, and labor‐saving benefits, especially in large‐scale surveys or studies involving large sample sizes, as demonstrated by spatiotemporal investigations of riverine biodiversity across Europe and North America (Perry et al. 2024). Because of these strengths, eDNA has been widely adopted in ecological studies, serving as an effective method for biomonitoring (Minamoto 2022).

Moreover, eDNA technology, in combination with scientific advances, has been applied to the investigation of target species, such as invasive (Tilapia) (Fang et al. 2025), endangered (Indo‐Pacific humpback dolphins) (Sun et al. 2025), and other rare taxa, biomass, and biodiversity (Perry et al. 2024; Wang et al. 2019). This method holds the potential to revolutionize assessments of ecological health for environmental monitoring. For instance, Suren et al. (2024) detected that eDNA can effectively complement traditional approaches by detecting patterns in invertebrate community composition, environmental responses, and biotic indices, despite occasional reference library gaps. For ecosystem health assessments, eDNA‐derived data on environmental indicator organisms can be utilized to construct databases that inform evaluation methodologies (Jiang et al. 2024). Furthermore, this technology allows for the exploration of organism community structures, thereby reflecting ecosystem health (Kim, Cho, Kim, et al. 2024). In summary, water contamination and the global spread of pathogens pose unexpected and significant challenges to human and environmental health (Liao et al. 2020). However, recent developments in DNA‐based technologies demonstrate new opportunities to address these challenges in areas such as biodiversity monitoring, ecosystem health assessment, and ecosystem restoration.

Environmental DNA technology is a versatile and powerful tool for addressing key biological questions regarding species diversity, distribution, and ecosystem health. Although eDNA technology shows many advantages, it also brings some challenges that need to be considered and solved (Joydas et al. 2024). For example, the generation and stability of eDNA vary greatly among taxa, individuals, and even tissues within the same organism (Bhendarkar and Rodriguez‐Ezpeleta 2024). There is a lack of comprehensive reference databases for a large number of taxa in eDNA analysis, and it is difficult to quantify species abundance and biomass (Beng and Corlett 2020). And contamination or incomplete DNA degradation can lead to false positives, while low eDNA concentrations or poor primer specificity can result in false negatives. Nevertheless, with the continuous progress and innovation of technology, eDNA technology is expected to be further optimized and improved through the combination of new technologies (Keck et al. 2023). Moreover, it can also promote the innovation and development of eDNA technology and expand its application scope through interdisciplinary cooperation and integration (Dumoulin et al. 2024). Therefore, eDNA technology is expected to be more widely used and developed in the future.

In summary, this review focuses on the breakthroughs in eDNA technology over the past 15 years and uses analytical software to present complex research results in a clear and intuitive manner. Then, the review summarizes the collection, detection, and analysis techniques of DNA in water, soil, and air environments. Based on a substantial body of literature resources, the review also systematically summarizes the wide application of eDNA technology in the key areas of scientific assessment of biodiversity, effective monitoring of invasive species and protection of endangered species. Despite these advancements, the practical application of eDNA still faces critical challenges, such as reference database gaps, risks to full‐process quality control and limitations in accurate abundance quantification. Lastly, the review proposes a forward‐looking development path for eDNA technology with constructive suggestions, which aligns closely with the trends of the current scientific and technological development.

## 2 Literature Analysis Methodology

### 2.1 Data Collection and Processing

The Web of Science (WOS) core collection database was used to conduct data collection, and the two topics “environmental DNA” and “biodiversity” were selected for the search process. As a result, a total of 3696 papers were retrieved from January 1, 2010, to December 1, 2025. Following a preliminary document type filter (Research Article and Review Article), 3638 documents remained, from which the top 1000 articles were selected based on WOS relevance to ensure robust clustering results that identify core research hotspots and frontier branches. Then, these articles were exported in plain text files. Specifically, the database file containing the 1000 articles was first exported in “Plain text file,” with the record content set to the “full record and cited references.” The downloaded file was then renamed in the format of “download_XXX.txt” before final export. For subsequent analysis using CiteSpace, four folders were created, namely input, output, project, and data. The “input” folder stores the text data, which is processed by the software and then exported to the “output” folder. The “data” folder holds the output data ready for analysis, and the “project” folder is used to display the results of running the analysis process software (Maheshwari et al. 2021). The co‐occurrence network and co‐citation analyses were clustered for evaluation and data visualization.

### 2.2 Analysis Methods

CiteSpace analyzes bibliographic records to model knowledge structures in a domain through time‐series networks from annual publications, which supports various bibliometric studies. It can help readers understand and systematically review the relevant literature. In this study, we focused on keyword clustering and literature co‐citation analysis from 2010 to 2025. Subsequently, a comprehensive analysis of the database was conducted, including keyword eDNA, timeline view of keyword “eDNA,” and co‐cited literature analyses. This helped identify core content and popular topics, as well as the development and evolution of each research cluster.

The analysis used CiteSpace 6.3.R1, with settings from 2010 to 2025, each year as a slice. Node types were keywords and references, with a Top N threshold of 30. This set up ensured all relevant literature data were included in the visualization network. CiteSpace's clustering depicted scientific knowledge graphically, where nodes represent items like keywords or references, and links show co‐occurrence or co‐citation. Node colors indicate age, with darker (purple, red) nodes representing earlier articles in the field.

### 2.3 Software Analysis Results

#### 2.3.1 Cluster Analysis of Keywords

**FIGURE 1:**
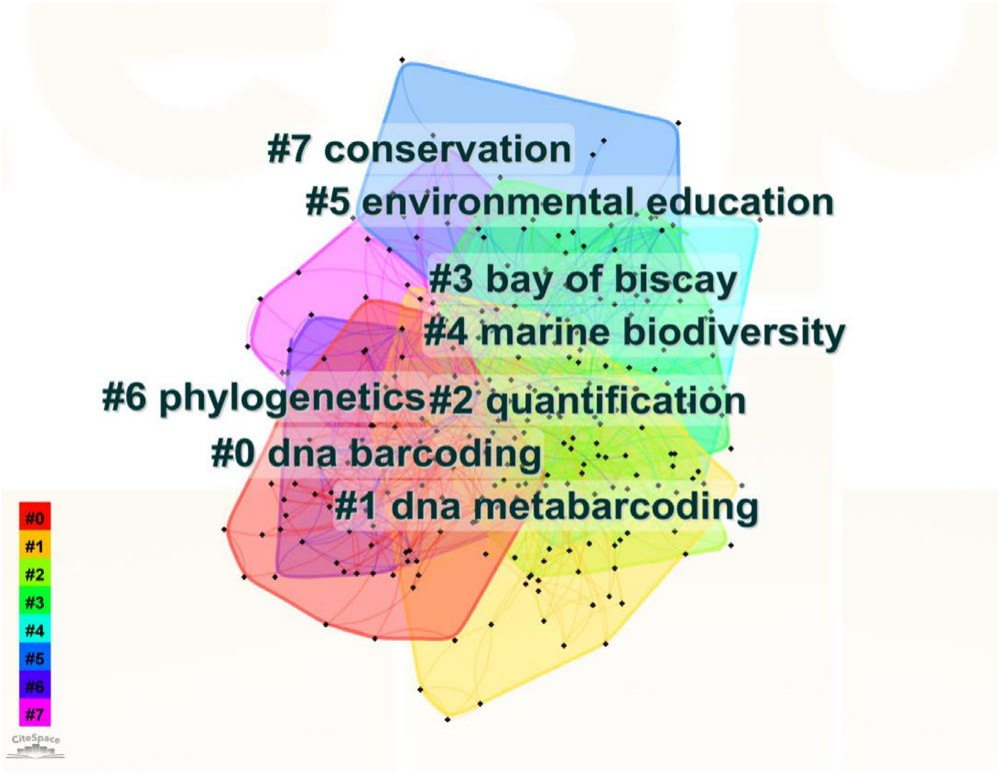
Keyword cluster analysis related to eDNA and biodiversity.

“Keyword cluster” analysis identifies the core content of publications in a highly generalized and condensed manner, indicating popular topics in a research field (Radhakrishnan et al. 2017). Figure 1 shows the cluster analysis results based on the keywords “eDNA” and “biodiversity.” As shown in Figure 1, clusters were obtained according to keyword co‐occurrence analysis, and a point or node represents an article. Different colors represent different clustering research directions. The smaller the number in the clustering diagram, the higher the frequency of occurrence of that cluster. The clusters “#0 dna barcoding,” “#1 dna metabarcoding” serve as the core methodology in this field, with such molecular techniques forming the fundamental tools for research. “#4 marine biodiversity” has been the primary research subject over the past 15 years. Relevant research has been conducted in typical regional scenarios such as the “#3 bay of biscay.” Indeed, the eDNA molecular technology extends its development toward “#2 quantification,” “#5 environmental education,” “#6 phylogenetics,” and “#7 conservation.” The clusters between molecular technology clustering and core research subjects demonstrate that molecular barcoding/metabarcoding technology has become an essential tool for marine biodiversity research, with its application gradually expanding from technical operations to societal applications. However, this figure highlights the mature application of eDNA technology in aquatic ecosystems. Notably, the absence of keyword clustering signals for tropical and terrestrial ecosystems indicates that research in these areas remains relatively underdeveloped.

**FIGURE 2:**
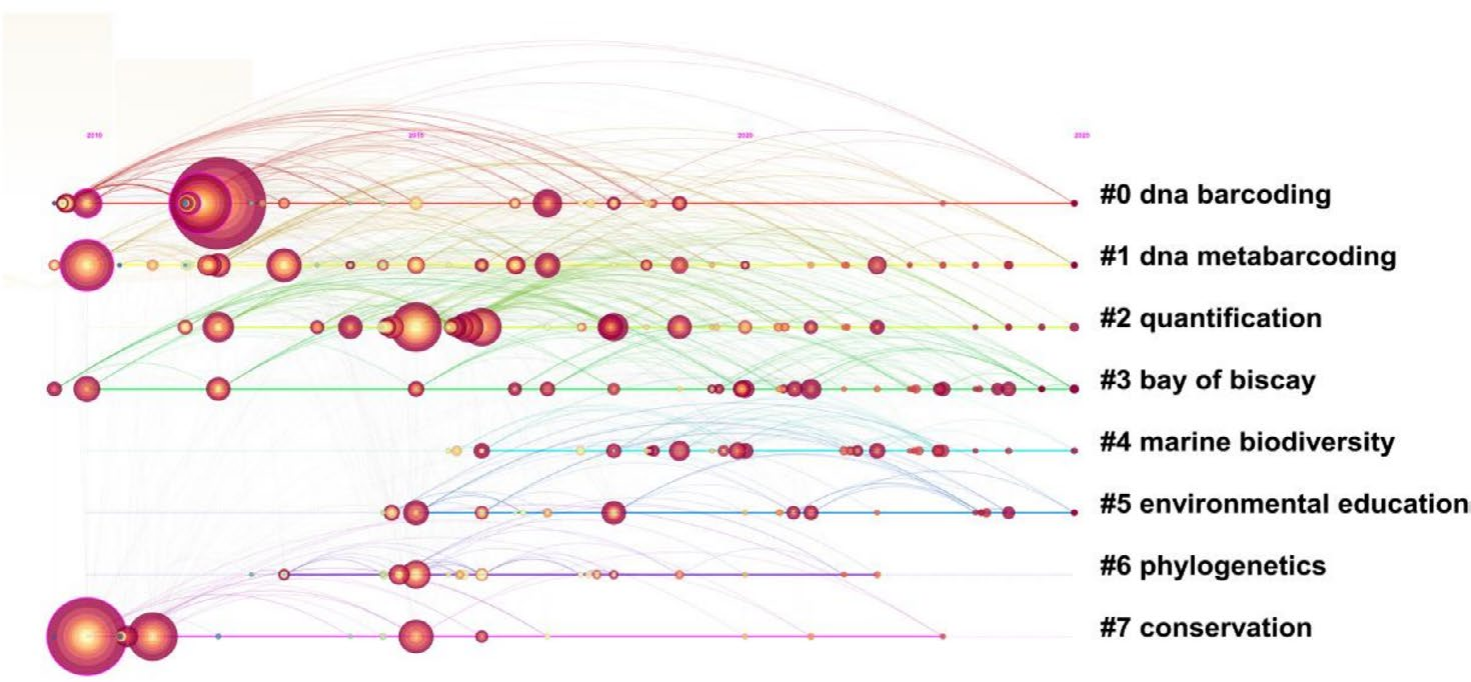
Timeline view of keyword eDNA and biodiversity.

The timeline view illustrates the development and evolution of each cluster's time span and research process. In this study, a red circle marks the emergence time of a keyword. Figure 2 presents a timeline view for the keywords “eDNA” and “biodiversity,” measuring their usage frequency change rate. This helps identify keywords that quickly gain widespread attention and become common research hotspots, guiding disciplinary development. The area of the circle in Figure 2 represents the number of studies published at that time and shows the 15‐year history of eDNA research. By analyzing the changes in the timeline of keywords, hotspots and trends in the research field can be identified. As observed in Figure 2, the keywords are more concerned by researchers in the related fields in the past 15 years. 2010–2013 is the hot period in the direction of DNA barcoding, DNA metabarcoding. Concurrently, conservation also saw significant research momentum, reflecting that this application goal was firmly established from the outset of the field's development. DNA metabarcoding exhibited a notable expansion in node proliferation and denser network connectivity after 2017, indicating its gradual replacement of traditional DNA barcoding as the mainstream technology in the field. And nodes related to quantification and phylogenetics became active, reflecting a shift in research focus from “species identification” to deeper “data quantification” and “evolutionary analysis.” Nodes related to marine biodiversity continued to cluster intensely after 2021, reflecting research focus in specific regions. The increase in nodes related to environmental education indicates that this field is expanding into science popularization applications. Over the past 15 years, research on aquatic ecosystems has centered on DNA barcoding and DNA metabarcoding as core technologies. However, research in other ecosystems remains relatively weak, consistent with the results shown in the clustering diagram.

#### 2.3.2 Co‐Citations Based on References

**FIGURE 3:**
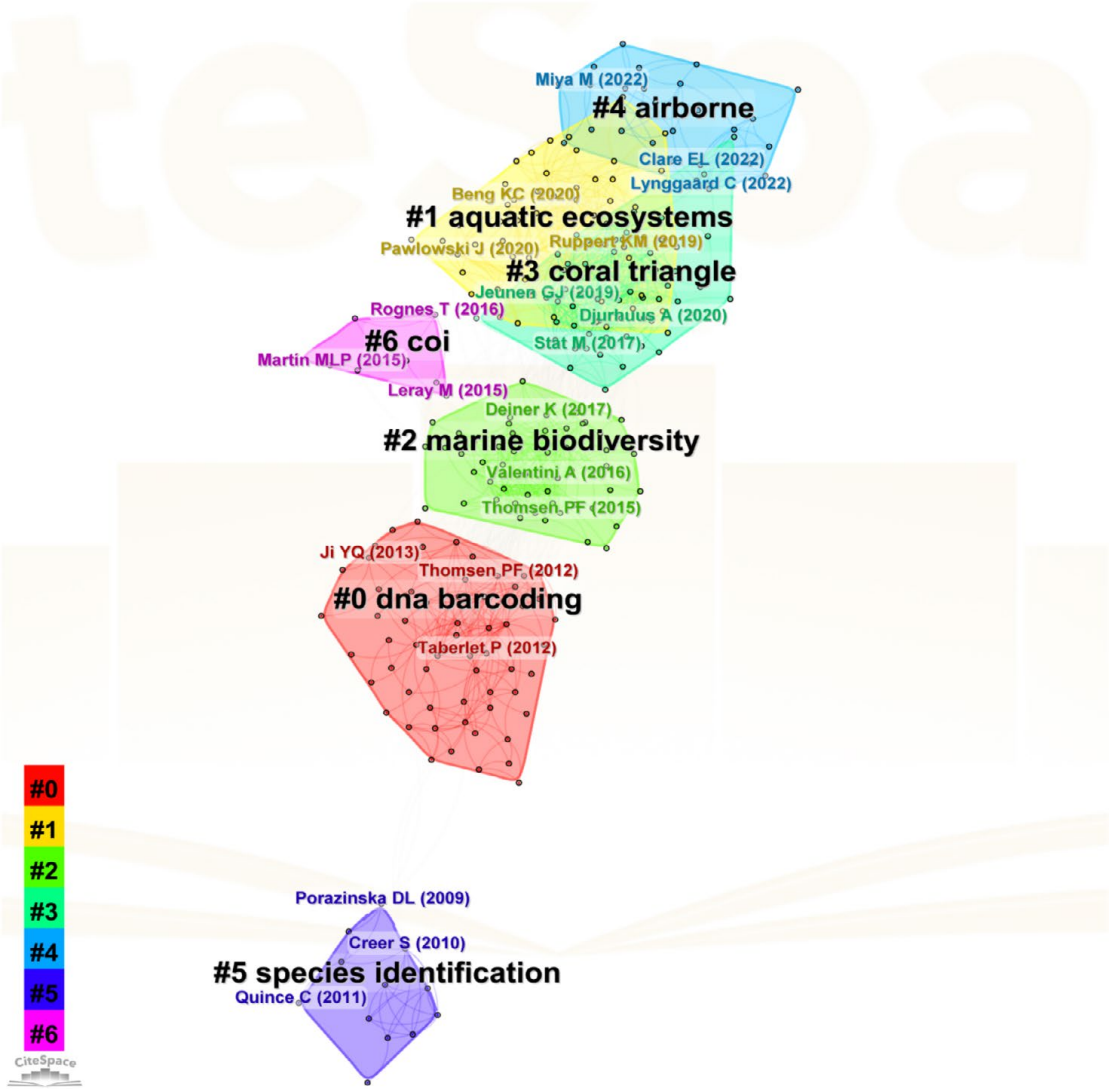
Co‐cited literature analysis on eDNA and biodiversity.

Figure 3 presents an analysis map of co‐cited literature. Co‐citation literature shares references with the present article, indicating common research content. More co‐citations imply a stronger article correlation. The co‐citation network's clustering location and relationships reveal the field's knowledge structure, providing an overall field understanding. This analysis identifies highly cited papers and influential literature, and through the co‐citation network, it also pinpoints similar literature's research topics and subject classification. This points in Figure 3 represent an article, while the color of the cluster indicates different citation frequencies. The smaller the number of clusters, the higher the frequency of co‐citation of research directions. Figure 3 shows DNA barcoding technology and COI establish the foundational technology. These technologies are applied to species identification and further extensively utilized in the fields of marine biodiversity and aquatic ecosystems. Simultaneously, research domains have expanded to coral triangle (a representative marine region) and airborne (another important environmental medium).

## 3 Research Progress on the Application of eDNA Technology in Monitoring Biodiversity and Assessing Ecosystem Health Based on Literature Statistics

### 3.1 Progress in Research Methods of eDNA Technology

#### 3.1.1 Water Sample Analysis

Environmental DNA technology has garnered extensive application in aquatic environments, emerging as a robust methodology for elucidating the biodiversity and biological characteristics of aquatic organisms (Elías‐Gutiérrez et al. 2021). The detection of eDNA in water bodies is principally compartmentalized into three sequential procedures: (1) the procurement and preservation of water samples; (2) the subsequent extraction of eDNA from these samples; (3) the amplification and analysis of the extracted eDNA to glean insights into the presence and nature of the organisms within the aquatic ecosystem (Zhang et al. 2023).

Water sample collection and preservation protocols vary across studies, and the following outlines commonly used methods in recent research (Table 1). Before sampling, all equipment are sterilized with ultrapure water and 75% alcohol, and sampling supplies need to be rinsed near the sampling point to prevent contamination (Jia et al. 2022). Sample volumes vary from 15 mL (e.g., laboratory tanks) to 10 L (e.g., lakes). Additionally, most samples collected in the past were around 1–2 L from streams, rivers, lakes, and seawater (Rees et al. 2014), but now many studies opt for large‐volume (e.g., > 5 L) filtration to minimize experimental variability (Le Guen et al. 2025). For small‐volume sampling, a sterile 60 mL Luer lock syringe can be used (Holman et al. 2019), while deeper water sampling often requires Niskin bottles or samplers (rosette samplers) (Zhang et al. 2023). After collection, water is stored in Nalgene polyethylene bottles at 4°C and filtered using a vacuum pump on the same day (Pilliod et al. 2013; Zhou et al. 2022). Filtration is typically performed using membrane pore sizes ranging from 0.2 to 0.45 μm, with 0.22 or 0.45 μm commonly used for microbial eDNA studies (Urycki et al. 2024). Moreover, glass fiber and cellulose acetate filter membranes demonstrate good capture efficiency for eDNA (Ding et al. 2025). After filtration, the membrane is folded and stored in a sterile centrifuge tube at −80°C (How et al. 2024).

**TABLE 1:** eDNA research methods for different environmental media.

	Water	Soil	Air
Sample collection and preservation	Luer lock syringe, rosette samplers; 4°C, membrane: −80°C	Corers, grabs, soil augers; −80°C	Geotech, MD8 airport sampler, air DNA ampler; membrane: −80°C
Environmental DNA extraction	DNeasy Blood and Tissue Kit; Power Water DNA Isolation Kit	DNeasy Power Soil DNA Isolation Kit	
Amplification	Metazoan (COI); plants (rbcL or ITS); eukaryotes (18S rRNA); prokaryotes (16S rRNA)		

The extraction of eDNA is a critical step in the analysis process, involving the use of proteases and surfactants to lyse cells and release DNA, followed by purification to separate DNA from other cellular components (Urycki et al. 2024). Numerous DNA extraction methodologies have been established, with several commercially available kits on the market. Among the most commonly employed are the DNeasy Blood and Tissue Kit (Qiagen) (Spens et al. 2017), PowerWater DNA Isolation Kit, Quick‐gDNA Centrifuge Column Kit (Wang et al. 2021), QIAamp DNA Microextraction Kit (Piaggio et al. 2014). A functional comparison of mainstream test kits is presented in Table 2. These kits offer standardized protocols that ensure consistency and reproducibility in eDNA extraction. In addition to commercial kits, the phenol‐chloroform‐isopentyl (PCI) alcohol DNA extraction method is also extensively utilized. This method is often paired with a room temperature preservation buffer, which provides the advantages of cost‐effectiveness and the ability to transport and store samples without the need for temperature control. This makes it particularly suitable for fieldwork and studies in remote locations. Following the initial extraction, further purification and concentration of eDNA can be achieved using disposable microfluidic devices. These devices enhance the precision of downstream assays by removing impurities and concentrating the DNA, thereby improving the sensitivity and accuracy of subsequent analyses (Carvalho et al. 2021).

**TABLE 2:** eDNA workflow performance comparison.

Extraction method	Representative kit/method	Suitable medium	DNA purity	Sensitivity	Buffer solution	References
Spin‐column based kits	DNeasy PowerWater Kit	Water*; air; soil	High	High, stable recycling efficiency.	EB buffer	Stojan et al. (2023)
	DNeasy Blood & Tissue Kit	Pure water	Extremely high	High	AE buffer	Chen et al. (2021)
	DNeasy PowerSoil Pro Kit	Soil*; water; air	Extremely high	High. High efficiency in breaking microbial cell walls.	EB buffer	Wang, Pijl, et al. (2024), Wang, Wang, et al. (2024)
	FastDNA SPIN Kit for Soil	Soil*; water; air	High	Extremely high. High efficiency in intense mechanical pulping.	TE buffer	Shi et al. (2022)
Magnetic bead based kits	MagMAX Microbiome Kit	Water*; air; soil	Extremely high	Extremely high. Most suitable for low‐biomass samples.	Elution buffer	Lau et al. (2023)
	NucleoMag DNA/RNA Water Kit	Water*; air; soil	High	High. Strong magnetic bead binding force, stable recovery.	TE buffer	Câmara dos Reis et al. (2023)
Precipitation/CTAB based methods	Laboratory‐prepared CTAB/phenol‐chloroform method	Soil*; water; air	Variable, typically poor	Variable. Poor repeatability.	Longmire's buffer, ethanol	García et al. (2024)

*Note:* * represents the most suitable medium.

Finally, for the amplification and analysis of eDNA, primers are crucial for targeting and amplifying specific DNA sequences via PCR (Tables 1 and 3). For quantitative species analysis, the use of highly specific primers and TaqMan probes is essential (e.g., quantitative PCR or droplet digital PCR) to ensure the exclusive amplification of the target species' DNA, thereby minimizing cross‐reactivity with nontarget species (Livak and Schmittgen 2001). In contrast, PCR aims for broad taxonomic detection while maintaining phylogenetic specificity. Therefore, the selection of primers is crucial. For post‐haplogroups, mitochondrial gene cytochrome oxidase I (COI) fragments are preferred (Adams et al. 2023; Sengupta et al. 2019). In plants, ribulose bisphosphate carboxylase (rbcL) and internal transcribed spacer region (ITS) genes are commonly amplified (Chase et al. 2020). For protozoa, 18S rRNA genes (V9 fragments) are selected, and for bacteria, 16S rRNA genes are used (Walters et al. 2016). The primers specific for amplifying other species are summarized in Table 3. After PCR, amplicons are assembled into a library and analyzed using next‐generation sequencing (NGS) technology for purification and sequencing.

**TABLE 3:** Summary of commonly used eDNA amplification sequences for research.

Target species	Amplified sequence	References
Vertebrate	12S 12SV05 F: TTAGATACCCCACTATGC 12SV05 R: TAGAACAGGCTCCTCTAG	Lynggaard et al. (2022)
Metazoan	CO1 mlCOIntF: GGWACWGGWTGAACWGTWTAYCCYCC jgHCO2198: TANACYTCNGGRTGNCCRAARAAYCA	Leray et al. (2013)
Fish	12S rRNA V12S‐U‐F: GTGCCAGCNRCCGCGGTYANAC V12S‐U‐R:ATAGTRGGGTATCTAATCCYAGT	Wang, Liu, et al. (2023), Wang, Wan, and Qian (2023)
Terrestrial plants	rbcL rbcLbF: AGACCTWTTTGAAGAAGGTTCWGT rbcLbR: TCGGTYAGAGCRGGCATRTGCCA	Liu et al. (2023)
Plankton	18S rRNA V9 1380F: TCCCTGCCHTTTGTACACAC 1510R: CCTTCYGCAGGTTCACCTAC	Liu et al. (2019)
Bacteria	16S V3‐V4 341F: CCTAYGGGRBGCASCAG 806R: GGACTACNNGGGTWTCTAAT	Huang et al. (2025)
Fungi	ITS1 1F: CTTGGTCATTTAGAGGAAGTAA 2R: GCTGCGTTCTTCATCGATGC	Abrego et al. (2024)

#### 3.1.2 Soil Sample Analysis

Today, many studies have also realized the potential of soil DNA in probing soil microbial diversity (Griffiths et al. 2000), but most studies have focused on studying aquatic ecosystems, using sediment eDNA to explore bioindicators associated with benthic macroinvertebrates, aquatic organisms, and physical and chemical factors (Pawlowski et al. 2022). Soil DNA extraction studies should follow the procedures outlined for the above water samples (Wei et al. 2018).

Initially, soil samples can be procured utilizing a corer or soil auger (Kakirde et al. 2010), while sediment collection can be accomplished with grabs (Turner et al. 2015). Alternatively, for shallow water sediments, sterile medicine spoons can be employed for direct collection. The ideal volume of a soil sample is contingent upon the size and biomass of the target taxa; for instance, a diminutive soil volume ranging from 0.2 to 0.5 g is adequate for assessing microbial diversity (Xie et al. 2017). Following collection, samples can be stored in sterile plastic bags or centrifuge tubes and brought back to the laboratory as soon as possible at low temperatures (Guerrieri et al. 2021). It is then stored in an ultralow temperature refrigerator at −80°C, which is the most reliable method of long‐term storage in current research (Pulido Barriga et al. 2025). To mitigate cross‐contamination between sampling stations, equipment should be rinsed with DNase‐free sterile water (Holman et al. 2019).

Second, for the soil DNA extraction process, the DNeasy PowerSoil DNA Isolation Kit (QIAGEN) is widely utilized for extracting eDNA from 0.25 g of soil (Thomson‐Laing et al. 2022; Table 2). Alternatively, for larger soil samples (4–8 g), the phenol‐chloroform‐isoamyl alcohol (PCI) method can be employed for DNA isolation, with the quality of the isolated DNA assessed via agarose gel electrophoresis using a 0.8% gel (Xie et al. 2017). Subsequently, during the amplification analyses of soil DNA, the selection of target genes depends on the specific research focus. The selection of amplification sequences should refer to the research methods for soil DNA described above. Following PCR amplification, the resulting products are prepared for sequencing using an appropriate high‐throughput sequencer. Then, generated sequences are subjected to bioinformatic analysis, including quality filtering, alignment, and taxonomic classification, to derive meaningful insights into the biodiversity and composition of the sampled soil environment (Xie et al. 2018).

#### 3.1.3 Air Sample Analysis

Most studies have been analyzing different sources of DNA in water, sediments, or soil (Stokstad 2021). Air is a medium for the dissemination of eDNA carried in bioaerosols, but the atmosphere as a source of genetic material encompassing all domains of life is mostly unexplored (Métris and Métris 2023). In existing studies, air DNA extraction can also follow the three steps outlined above for water and soil samples (Goray et al. 2024).

For air DNA collection, there are two primary methods for collecting airborne DNA: active and passive sampling methods. Among these, passive sampling involves directly fixing filter membranes in the field to collect DNA (Lin et al. 2025). However, this method cannot quantitatively collect samples. Therefore, current research primarily utilizes active air filtration machines. For example, air samples are procured utilizing a peristaltic pump (Geotech), with a filter (Merck Millipore) inserted at the hose's inlet. This setup facilitates the external suction of air, which is then directed through the filter for collection (Clare et al. 2022). After sample collection, all filters were placed in sterile bags and frozen until DNA was extracted (Bohmann and Lynggaard 2023). In addition to common filters, researchers have also invented filter‐based air samplers such as the MD8 Airport and AirDNA sampler (Harnpicharnchai et al. 2023). These air samplers enhance the collection of bioaerosols by employing simple, cost‐effective portable ventilation fans coupled with customized multi‐filter holders. This approach provides a convenient and efficient means of obtaining high DNA yields from bioaerosols, which are subsequently amenable to precise and reliable molecular analyses, encompassing metabarcoding and macro‐genome sequencing.

In the amplification of airborne DNA, it is imperative that all tools and instruments are sterilized using UV light and cleaned with 10% bleach and 70% ethanol between samples to prevent cross‐contamination (Cruaud et al. 2017). In most studies, DNA was extracted using Qiagen DNA Blood and Tissue kits (Lynggaard et al. 2022; Table 2). Finally, the same principles apply to airborne DNA amplification as to soil DNA. Following amplification, the integrity and quality of the PCR products can be assessed by visualizing them on a 2% agarose gel (Banchi et al. 2020), Thereafter, the amplicons are subjected to sequencing, and the resultant sequences are analyzed using bioinformatics tools to interpret the data and draw meaningful conclusions (Garrett et al. 2023).

### 3.2 Optimized Pathways and Critical Challenges in eDNA Analysis

Although molecular biology tools are widely used in eDNA extraction, standardized protocols are not universally applicable, as method performance, cost, and applicability vary among extraction kits, preservation buffers, and primer sets. Researchers should carefully weigh these factors based on specific research objectives and conditions. This work summarizes common trade‐offs among different workflow components in terms of key performance metrics in Tables 2 and 3. In addition, targeted recommendations are lacking in current research for critical workflow nodes, such as optimizing biomass estimation methods (e.g., occupancy models, ddPCR/qPCR selection), establishing reference database standards, and defining reference database criteria (e.g., taxonomic coverage gaps, local barcoding needs, mis‐annotation rates, and proposed minimum reporting standards). Therefore, we focus on these critical aspects in the following sections.

#### 3.2.1 The Challenge of Bioinformatic Analysis and Big Data Management

The species identification capability of eDNA technology fundamentally depends on the coverage and accuracy of the reference database. The International Nucleotide Sequence Database Collaboration (INSDC, http://www.insdc.org), one of the most enduring worldwide alliances of biological data repositories, is dedicated to capturing, preserving, and providing open access to comprehensive nucleotide sequence information in the public domain. Through close collaboration, the three partner organizations of INSDC develop unified data and metadata standards, as well as submission protocols, to ensure reliable data deposition into their respective databases and enable seamless global data exchange. The INSDC consists of three partners: the DNA Data Bank of Japan (DDBJ, http://www.ddbj.nig.ac.jp/) at National Institute for Genetics in Mishima, Japan; the European Molecular Biology Laboratory's European Bioinformatics Institute (EMBL‐EBl: http://www.ebi.ac.uk/ena) in Hinxton, UK, and the National Center for Biotechnology Information (NCBI, http://www.ncbi.nlm.nih.gov/genbank/) in Bethesda, USA (Karsch‐Mizrachi et al. 2017). Among these, NCBI is the most widely used in eDNA metabarcoding studies, primarily owing to its comprehensive sequence resources (e.g., Genbank) and user‐friendly analytical tools. For instance, a soil biodiversity study leveraged NCBI's databases to assign eDNA sequences across multiple trophic levels, from fungi and bacteria to metazoan and other eukaryote, revealing the ecological health of agroecosystems (Xing et al. 2024). Similarly, NCBI reference data were instrumental in a riverine eDNA survey that simultaneously characterized community structure from primary producers (algae) to top predators (fish), providing a holistic ecosystem assessment (Li et al. 2025).

However, the publicly accessible reference repositories (primary or secondary) are not always the optimal solution for several reasons. For instance, regarding species coverage, some rare or understudied taxa (e.g., *Ethmalosa fimbriata* ) remain difficult to annotate (Cox et al. 2024; Nneji et al. 2025). Additionally, geographic biases in data distribution also pose limitations, such as most public database data coming from temperate freshwater ecosystems, leaving gaps for tropical freshwater fish (e.g., Amazon Basin) (Ruiz‐Tafur et al. 2024). Consequently, many research institutes compare eDNA sequences against both public open databases and a curated study‐specific (closed) reference database. For example, “AeDNA” is a specialized aquatic eDNA database designed to provide comprehensive reference sequences for aquatic biodiversity surveys (Chen et al. 2022). Thus, integrating researcher‐built and closed databases with public repositories, as well as enhancing the standardization of analytical workflows and quality criteria for these datasets, will be of great significance for advancing eDNA analysis and research.

#### 3.2.2 The Challenge of Moving From Qualitative to Quantitative

While eDNA is valued as a minimally or noninvasive tool and is increasingly employed for qualitative species detection, the quantitative conversion of species abundance remains a core issue in the field. Thus, we synthesized the principal approaches now delivering quantitative, reproducible estimates. First, because species are rarely detected perfectly at all truly occupied sites during surveys (false negatives), occupancy models account for imperfect detection and are thus especially useful for eDNA surveys, considering DNA is not observed or may go uncollected when in fact present during sample collection. These models have been increasingly utilized in eDNA studies (Tetzlaff et al. 2024). The application of such models in eDNA research is increasingly widespread. Moreover, this model has been applied to the monitoring of rare, highly mobile, and inconspicuous species such as *Gulo gulo* and *Ochotona princeps* (Emmet et al. 2021; Goldman et al. 2023). Furthermore, droplet digital PCR (ddPCR) is a novel nucleic acid quantification technique that divides DNA samples into thousands of independent droplets, performing PCR reactions within each droplet. By counting the number of positive droplets, ddPCR directly calculates the absolute copy number of target DNA without relying on standard curves. Compared to qPCR, ddPCR offers higher sensitivity and precision, making it particularly suitable for detecting low‐abundance target DNA and analyzing complex environmental samples (Guri et al. 2024). It enables precise and sensitive quantitative analysis of eDNA, facilitating the tracking of rare (e.g., *Haliotis kamtschatkana* ) (Dimond et al. 2022) and invasive species (e.g., *Lithobates catesbeianus* ) (Everts et al. 2021). Currently, this technology also remains predominantly applied in aquatic ecosystems. Finally, especially in dynamic aquatic ecosystems, the transport and decay processes of eDNA significantly impact signal tracing and quantitative accuracy. Therefore, coupling hydrodynamic transport or decay models with eDNA concentration data has become a crucial method for back‐calculating biomass distribution at the watershed scale, pinpointing pollution sources, or locating endangered species (Carraro et al. 2023). Systematically integrating these diverse approaches will be the core direction for achieving precise quantitative applications of eDNA in biodiversity monitoring and ecosystem management in the future.

#### 3.2.3 Challenges in Full‐Process Quality Control

Throughout the entire process, eDNA results also depend on the effective control of false negatives (where the target species is present but not detected) and false positives (where the target species is absent but erroneously detected). To avoid false negatives, the core lies in ensuring complete capture and efficient detection of eDNA. Key measures include: optimizing sampling design (e.g., using fine‐mesh filters, collecting large‐volume water samples); maintaining low‐temperature storage throughout to prevent DNA degradation; and employing qPCR and ddPCR technologies to enhance detection sensitivity (Pinfield et al. 2019). For false positives, the core lies in rigorously preventing contamination and misinterpretation. Strict anti‐contamination protocols must be established, including physical isolation of experimental areas, use of sterile consumables, and thorough disinfection of equipment. More importantly, multiple blank controls (e.g., field blanks, extraction blanks, PCR blanks) should be systematically implemented to identify and eliminate contamination sources. During bioinformatics analysis, false signals arising from contamination or sequencing errors must be filtered out by setting thresholds for sequence similarity and minimum sequencing depth (Ficetola et al. 2016). In summary, implementing this integrated quality control system, combining process controls, blank controls, and bioinformatics filtering, is fundamental to ensuring the scientific validity and reliability of eDNA research outcomes.

### 3.3 Research Progress on the Application of eDNA Technology in Monitoring Biodiversity

Biodiversity includes three levels: genetic diversity, species diversity, and ecosystem diversity. Biodiversity reflects the richness of life forms and processes on the planet, and its value is immeasurable. It not only constitutes the natural foundation of life activities (Larsen 2024), but also is the key to maintaining the ecological balance of the earth. However, the loss of a species or the introduction of invasive species can disrupt this delicate equilibrium, leading to cascading effects on ecosystem stability. Consequently, monitoring and managing biodiversity are crucial (Pennekamp et al. 2018). Extensive international research has demonstrated the effectiveness and sensitivity of eDNA technology in ecological monitoring, establishing it as a useful tool for biodiversity assessment (Evans et al. 2016). Currently, the application of eDNA technology in China mainly includes monitoring of common species, rare and endangered species, and invasive species. eDNA technology is versatile and can be used to retrieve information on aquatic, semiaquatic, and terrestrial species. By analyzing DNA fragments shed into the environment, eDNA enables noninvasive, efficient, and accurate detection of species presence, making it convenient and practical for modern biodiversity research and conservation efforts.

According to a massive new UN‐sponsored report from the Intergovernmental Science‐Policy Platform on Biodiversity and Ecosystem Services (IPBES), the four sections of the summary cover the decline in biodiversity and ecosystem services, the accelerating drivers of this change, the failure to meet many of the goals set for conservation, and the very challenging actions needed to restore sustainability (Bongaarts 2019). In this context, the advancement of eDNA and sequencing technologies has garnered significant attention as a transformative approach for both targeted species detection and assessments of the biodiversity of any ecosystem (Sahu et al. 2023). Illustratively, the eDNA approach has demonstrated a capacity to detect twice the number of fish species compared to the conventional beam trawl method (Wu et al. 2024); the integration of eDNA with related sequencing technologies significantly outperforms traditional technologies in detecting species richness (Kasmi et al. 2024); a study has used the DNA metabarcoding method to evaluate biological communities, overcoming the limitations of morphological species identification (Kiemel et al. 2023).

### 3.4 Research Progress of eDNA Technology in Precious and Invasive Species

The analysis of eDNA is a powerful, nondestructive technique for detecting rare or hard to find organisms. Different countries and regions have their own rare species that are often difficult to find through traditional methods. For example, the golden tree frog ( *Phytotriades auratus* ) is a rare amphibian found only in *Glomeropitcairnia erectiflora* on the highest peaks of the island of Trinidad in the West Indies. Moreover, eDNA evidence from water samples obtained from waterholes in Australia suggests that the endangered Gouldian finches ( *Erythrura gouldiae* ) have visited the waterholes (Lewis 2019). Similarly, the uncommon Dwarf sperm whale ( *Kogia sima* ) has been detected around the remote Malpelo island in Colombia through eDNA analysis (Juhel et al. 2021). In another example, eDNA collected from 27 sites along the final stretch of the Yangtze River and the Yellow Sea coast was analyzed using quantitative PCR (qPCR) and eDNA metabarcoding, leading to the detection of the critically endangered Yangtze finless porpoise (Chen et al. 2024). These studies underscore the efficacy of eDNA techniques in identifying and monitoring rare species in diverse and challenging environments.

Under the combined influence of global warming and economic globalization, biological invasion has become one of the hot topics in the field of global ecology, posing significant threats to native ecosystems and biodiversity (Wang, Liu, et al. 2023; Wang, Wan, and Qian 2023). Currently, eDNA technology is widely used for monitoring aquatic invasive species (López‐González et al. 2025). For instance, eDNA techniques have been employed to track the invasion of crayfish across various European countries (King et al. 2022). Additionally, the red drum *Sciaenops ocellatus* , an invasive species in the East China Sea, has been detected using eDNA methods (Wang et al. 2022). Beyond aquatic ecosystems, eDNA technology has also been applied to monitor invasive terrestrial organisms. One study has demonstrated that eDNA analysis can serve as a valuable monitoring tool for alien ants, using *Linepithema humile* (Argentine ant), one of the most invasive ant species (Yasashimoto et al. 2021). Furthermore, eDNA technology has successfully detected the new invasion of the destructive New Zealand mud snail *Potamopyrgus antipodarum* (NZMS), highlighting its utility in early detection and management of invasive species (Woodell et al. 2021). However, most current research focuses on species monitoring in aquatic ecosystems, which reflects that there is still much room for the development of eDNA in terrestrial ecosystems. As research and methodological advancements continue to advance, eDNA techniques are expected to play an increasingly crucial role in global ecological surveillance, aiding in the protection of biodiversity.

### 3.5 Research Progress on the Application of eDNA Technology Assessing Ecosystem Health

**FIGURE 4:**
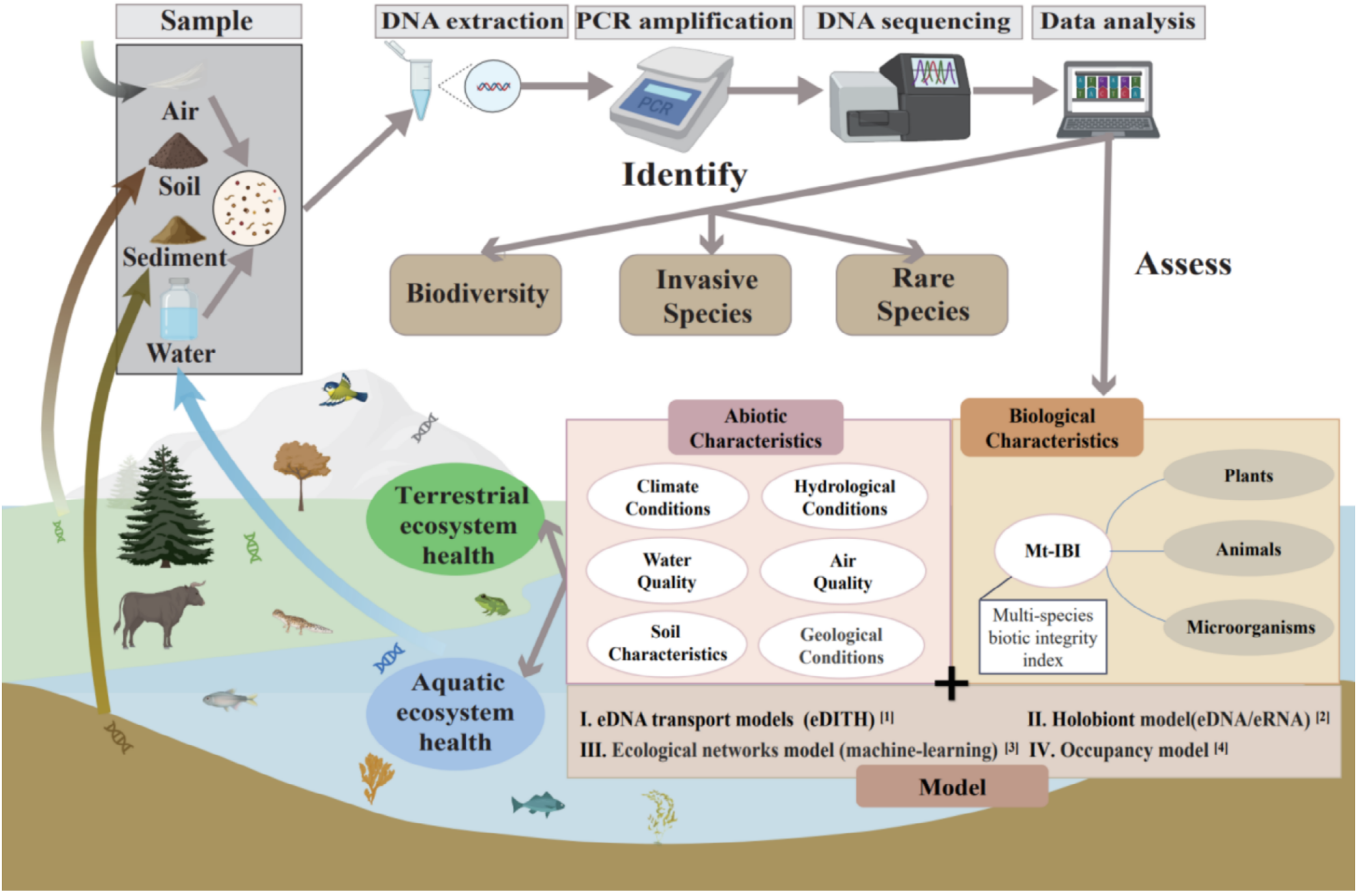
Schematic diagram of eDNA sample collection, analysis, and functions, with reference to representative studies (Bohan et al. 2017; Carraro et al. 2023; Cook et al. 2025; Rounds et al. 2024).

As the UN advances the post‐2020 global biodiversity framework under the Convention on Biological Diversity, emphasis is placed on aligning new ecosystem conservation goals with its vision of “living in harmony with nature” (Keith et al. 2022). Ecosystem health assessment plays a critical role in understanding the status of regional ecosystems, identifying environmental issues, and providing a scientific basis for formulating effective ecological protection strategies. Such assessments are essential for guiding sustainable management practices and enhancing environmental governance. In recent years, the rapid development of eDNA technology has significantly improved the monitoring capability of ecosystem health (Figure 4).

First, rapid and reliable monitoring and prediction of ecological change trends are of great value to the protection of aquatic ecosystems. eDNA technology can be used to detect species richness in ecosystems (Deiner et al. 2017), identify relevant indicator species, calculate ecosystem diversity indices, and analyze functional genes of soil microorganisms (Pawlowski et al. 2021) to assess ecosystem health. Looking at it from a comprehensive point of view, many innovative methods have been applied to the monitoring of aquatic ecosystem health. For example, in the assessment of aquatic ecosystem health based on the Index of Integrity (IBI), fish, benthic organisms, and living algae are often used as indicators. However, the number of samples available for assessment has become less, creating a wide application potential of the eDNA‐based Mt‐IBI method in the health assessment of river ecosystems (Song et al. 2025). Besides, the research showed that both qualitative and quantitative eDNA‐based biological indices were correlated with water quality seasonally by monitoring zooplankton structure. The season‐dependent eDNA zooplankton integrity index (IZI) reflected the ecological status, and this method improved the timeliness of the health of aquatic ecosystems bioassessment (Yang and Zhang 2020). Of particular concern is the integration of eDNA metabarcoding approaches with machine learning, remote sensing, and other interdisciplinary technologies that could enable more accurate and cost‐effective detection of aquatic ecosystem conditions. Traditional ecosystem health monitoring methods, which rely on direct observation and sampling of indicator species, are often labor‐intensive, time‐consuming, and limited in their ability to provide timely and comprehensive data, particularly in large or remote aquatic systems (Pinna et al. 2023). In contrast, eDNA technologies offer a more efficient and sensitive approach to monitoring. They detect the genetic signatures of various organisms from environmental samples, providing a broader, more detailed understanding of ecosystem health. In certain circumstances, this makes eDNA technology not only a complement to traditional methods, but also offers advantages over either approach when used in combination (Erős et al. 2025; Kim, Cho, Hwang, et al. 2024).

Environmental DNA technology has emerged as an important tool for assessing the health of terrestrial ecosystems, although research in this area remains significantly less extensive compared to aquatic ecosystems. By extracting and analyzing genetic material from environmental media such as soil, eDNA techniques provide researchers with a unique and efficient approach to evaluating biodiversity and ecosystem health. Specifically, eDNA analysis is particularly adept at revealing the presence of indicator species (e.g., nematodes) that are inherently sensitive to environmental changes, thereby serving as early warning signals for potential disturbances in ecosystem health (Carini et al. 2017). Furthermore, the strategic integration of eDNA data with other ecological indicators, such as vegetation cover and soil chemistry, significantly enhances the comprehensive evaluation of ecosystem health. Indeed, compared with traditional methods such as trapping or direct observation, this method is faster and more cost‐effective, so it can understand the ecosystem more comprehensively and expansively (Saenz‐Agudelo et al. 2022). In essence, eDNA technology represents a significant advancement in ecological monitoring, offering a powerful tool for the rapid, sensitive, and cost‐effective assessment of terrestrial ecosystem health (Banerjee et al. 2022).

## 4 Implication and Prospect

The rapid development of eDNA technology holds significant promise for addressing biodiversity and ecosystem health assessment. To maximize the potential of eDNA applications, future research could integrate this approach in the following critical directions to advance ecological monitoring and conservation efforts.

First, climate change impacts could leverage eDNA metabarcoding to track ecological responses. For instance, spatiotemporal analysis of biological communities through COI amplicon‐based forecasting models enables precise assessment of warming and acidification impacts (Gallego et al. 2020). Concurrently, eDNA with unified protocols for sample collection, storage, and bioinformatics will improve data comparability across different study scales and climate research projects.

Second, eDNA technology shows great potential for environmental pollutant monitoring. High‐throughput eDNA assays effectively detect marine pollutants (Rishan et al. 2024) and terrestrial contaminants via pollution‐sensitive bioindicators and functional gene markers. This approach addresses critical environmental challenges such as plastic waste and chemical runoff that threaten ecosystem biodiversity, stability, and human health (Zhang 2019).

Additionally, eDNA can be integrated with advanced technologies, including machine learning, remote sensing, and bioinformatics, to enhance ecological research. For example, integrating eDNA with machine learning‐assisted image recognition (e.g., arthropod distribution mapping via DNA barcoding + satellite imagery) (Li et al. 2024), remote sensing (e.g., UAV‐swabbed canopy eDNA) (Kirchgeorg et al. 2024), and automated sampling systems (e.g., robotic water samplers) (Wang, Pijl, et al. 2024; Wang, Wang, et al. 2024) could revolutionize real‐time ecosystem surveillance. Overall, these integrated approaches would promote environmental monitoring, enabling more precise, scalable, and cost‐effective conservation strategies.

## Author Contributions

**Shuwen Wu:** conceptualization (equal), software (lead), writing – original draft (lead), writing – review and editing (equal). **Yun Wang:** writing – original draft (supporting), writing – review and editing (equal). **Haiyan Qin:** writing – review and editing (supporting). **Zeyu Zhang:** writing – review and editing (supporting). **Shijun Liu:** writing – review and editing (supporting). **Yunjie Ruan:** supervision (equal), writing – review and editing (supporting). **Guangsuo Chen:** writing – review and editing (supporting). **Xia Yuan:** conceptualization (lead), funding acquisition (lead), resources (equal), supervision (lead), writing – original draft (equal), writing – review and editing (lead). **Hangjun Zhang:** conceptualization (equal), supervision (equal), writing – review and editing (equal).

## Funding

This work was supported by the “Pioneer” and “Leading Goose” R&D Program of Zhejiang (2024C03226).

## Conflicts of Interest

The authors declare no conflicts of interest.

## Supporting information


**download1‐1000:** ece372891‐sup‐0001‐download1‐1000.txt.

## Data Availability

The data that support the findings of this study are available in the Supporting Information (download1‐1000).
